# Anti-Asian Racism: Developing a Brief, Reliable, and Valid Measure

**DOI:** 10.1007/s40615-026-02939-7

**Published:** 2026-04-27

**Authors:** Daniel B. Lee, Xining Yang, Tsu-Yin Wu, Isaac Mouacheupao, Marc A. Zimmerman, Hsing-Fang Hsieh

**Affiliations:** 1 University of Michigan, Institute for Firearm Injury Prevention, Ann Arbor, MI, USA; 2 Department of Geography and Geology, Eastern Michigan University, Ypsilanti, MI, USA; 3 School of Nursing, Eastern Michigan University, Ypsilanti, MI, USA; 4 Carleton College, Northfield, MN, USA

**Keywords:** racial discrimination, measurement, psychometrics

## Abstract

**Objective:**

We evaluated the dimensionality of an Anti-Asian racism (AAR) measure, informed the development of a brief measure, and validated a brief AAR measure.

**Method:**

We conducted a national survey of 748 Asian American adults in 2023 (64.6% female). Bi-factor analysis was used to evaluate the dimensionality of the AAR measure, and Graded Response Models (GRM) informed the development of the brief measure of AAR. We also evaluated concurrent validity.

**Results:**

The AAR scale is unidimensional. GRM was conducted to derive a brief (6-items), reliable, and valid measure of AAR. Our brief measure of AAR yielded valid and reliable scores, and was associated with firearm purchasing, depressive symptoms, anxiety symptoms, and appraisals of safety, demonstrating concurrent validity.

**Conclusions:**

The brief measure of AAR includes items related to subtle, blatant, and cultural racism. The brief scale offers opportunities for real-time, momentary assessments and opportunities to discuss AAR in the community health setting.

Racism is a fundamental driver of health disparities [33, 44], and researchers have made great strides in measuring racism in racially minoritized communities [1, 12, 19, 32]. Asian Americans are no strangers to racial discrimination and, more recently, the COVID-19 pandemic exacerbated the prevalence and pervasiveness of this stressor. Anti-Asian racism (*hereafter*, AAR) – referring to discriminatory actions targeting Asian Americans due to prejudice and racialized stereotypes (e.g., blaming Asians for COVID-19; [9]) – invokes significant stress and undermines health [17, 21, 30]. Given the public health significance of AAR, researchers developed novel measures or adapted existing ones to assess this significant source of stress among Asian Americans [10, 29, 49]. However, a large proportion of these measures have focused on subtle forms of AAR (e.g., microaggressions), with less attention to blatant (e.g., racialized verbal or physical harassment) and cultural forms (e.g., negative portrayals of Asian Americans in the media). Moreover, many measures of racial discrimination for Asian Americans are lengthy (25 + items; e.g., Kwok et al., [25]. While high-item measures can be important for capturing the nuances of AAR, these measures may be less practical in settings (e.g., healthcare, community) where participants have limited time. This limitation can result in lower completion rates, non-response bias, and limit the ability to measure other constructs of interest. In contrast, brief measures may increase completion rates, enable real-time assessments (e.g., ecological momentary assessments [EMA]), and produce valid and reliable scores with fewer items. Thus, we developed and psychometrically validated a brief measure of AAR in a national sample of Asian Americans.

## The Multifaceted Nature of Anti-Asian Racism

Racism is multifaceted. Dr. James Jones seminal Tripartite Model of Racism elucidates three subtypes: (1) individual racism, (2) institutional racism, and (3) cultural racism [22]. Individual racism refers to experiences of racial discrimination that occur at the interpersonal level, and other researchers have distinguished two subtypes – that is, subtle and blatant racism [40]. Subtle racial discrimination involves experiencing indirect and ambiguous actions that marginalize individuals by race (e.g., racial microaggression), whereas blatant racial discrimination entails overt, explicit, and intentional acts of racial prejudice and bias (e.g., being called a racial slur). Institutional racism refers to the institutional arrangements of programs and policies that disadvantages one racial group while providing advantages to another (e.g., unfair policing practices; [22]). Lastly, cultural racism are practices that reinforce intergenerational ideologies that position racial groups as superior or inferior to another [22]. Other subtypes of racism have followed since the seminal conception of the tripartite model (e.g., vicarious racism; see [32] for review).

The preponderance of AAR measures evaluates interpersonal experiences, such as microaggressions and verbal harassment (e.g., [16, 49]). Yet, the COVID-19 pandemic facilitated a surge in other AAR experiences, including but not limited to cultural racism and blatant acts of interpersonal racism (e.g., physical attacks). For instance, since the onset of COVID-19, researchers documented an uptick in AAR that involved physical attacks and threats [18]. Asian Americans have also raised increasing concerns about cultural racism, largely driven by rhetoric used by politicians and mainstream media (e.g., *China Virus*, *Kung Flu*) to blame Asian Americans for COVID-19 [26]. The negative portrayal of Asian Americans as *foreigners* and *dirty* is a significant source of stress that ultimately undermines mental health [21]. While Asian Americans have long experienced AAR, even prior to COVID-19, the pandemic triggered a surge across the various types of AAR, including subtle, blatant, and cultural forms.

### Measures of Anti-Asian Racism

Multiple measures have been used to assess racism experiences among Asian Americans [16, 20, 25, 29, 49]. While these measures are crucial for evaluating racism as a determinant of health, two research gaps remain. First, guided by the Tripartite Model of Racism [22], racism is a multidimensional phenomenon encompassing experiences at the interpersonal, institutional, and cultural levels. While several measures of racial discrimination for Asian Americans assess multiple dimensions of racism—such as, the PEDQ-CV [4, 25] and the Subtle and Blatant Racism Scale for Asian American College Students (SABR-A^2^; [49]—the majority of these measures have primarily focused on interpersonal racism experiences (e.g., microaggressions, verbal harassment). To this end, much of the research on AAR has documented the health consequences of interpersonal racism [16, 27]. Asian Americans are, however, also vulnerable to other forms of racial discrimination such as cultural racism [48]. Therefore, it is essential for researchers to capture the full scope and severity of AAR as experienced by Asian Americans. This includes, but is not limited to, cultural racism, such as the portrayal of Asian Americans in a negative light in mainstream media.

Second, many measures of AAR are lengthy due to its multidimensional structure (i.e., measures that evaluate multiple subtypes of racism). While no formal benchmark defines what constitutes a lengthy measure, this can vary by project (e.g., incentive structure, cognitive load to complete items), researchers studying racism experiences among Asian Americans are often interested in assessing additional constructs related to theorized outcomes (e.g., mental health indicators), moderators (e.g., resilience-promotive factors), mediators (e.g., stress-related pathways), and control variables (e.g., sociodemographic correlates). As such, a brief measure of AAR may provide more space in surveys to include additional constructs of interest. Moreover, lengthy measures are not always practical for settings where data collection needs to be rapid (e.g., real-time data collection efforts) or when respondents have limited time (e.g., patients receiving treatment in an emergency department). Developing concise measures of AAR could facilitate research designs incorporating real-time data collection methods (e.g., ecological momentary assessments), allow space for additional constructs in surveys (e.g., moderators, mediators), and reduce respondent burden. This is particularly important for respondents from marginalized groups, where non-response bias is exceptionally high, underscoring the need for surveys that are not only culturally relevant and sensitive but also concise [5, 24]. Of note, while brief measures are often unidimensional, it is crucial that items capture the different dimensions of AAR to reflect its full scope. This ensures that the brief measure captures one’s overall experience of AAR, rather than a single subtype (e.g., microaggression).

### Current Study

We endeavored to develop and psychometrically validate a brief measure of AAR. The psychometric evaluation had three objectives: (1) explore the dimensionality of the AAR measure to assess if AAR supports reliable scoring for one or more subtypes, (2) develop a brief measure of AAR that yields reliable and valid scores, and (3) evaluate the reliability and convergent validity of the brief measure relative to the full measure. To assess concurrent validity, we investigated whether the brief and original AAR measures were similarly associated with previously documented psychological and behavioral outcomes: (1) safety concerns due to racism [51], (2) depressive symptoms [7, 43], (3) anxiety symptoms [21, 34], and (4) firearm purchasing [47].

## Method

### Participants

Participants were drawn from a cross-sectional, national survey of 748 Asian American adults conducted in July 2023. The online surveys were administered through the Dynata Simplify Panel, which comprises approximately 2.5 million U.S. residents. To ensure data reliability and accuracy, the Dynata team employed a rigorous verification process, including digital fingerprinting and spot-checking through third-party verification to confirm participant identity. Eligible participants were individuals aged 21 years or older who self-identified as Asian Americans, could read English, and had internet access via a computer or smartphone to complete the online survey. A total of 1,159 eligible participants were invited to participate, with 1,059 (91.3%) providing consent. After excluding responses that fell below the minimum survey completion time threshold (under 5 min, *n* = 373; i.e., not feasible to complete the survey in less than 5 min even when all skip patterns were applied) and those with complete missing data for the variables included in this study (*n* = 114), the final sample consisted of 748 participants with complete data. Informed consent was obtained electronically from all participants. The study protocol and procedures were approved by the Institutional Review Board of the first author’s institution (HUM00221554).

Of the 748 participants, 64.6% were female, and the majority had completed an undergraduate degree (62.83%) or a graduate degree (28.98%). The participants’ age was assessed in ordered categories (i.e., [21-24] to [85+]); on average, participants fell between the 35–44 and 45–54 age groups (M = 2.74, SD = 1.71). In terms of ethnicity, Chinese Americans (23.1%) comprised the largest ethnic group in the analytic sample, followed by Filipino (19.6%), Indian (19.5%), Japanese (11.9%), Korean (6.3%), Vietnamese (5.7%), and mixed Asian ethnicities (4.6%). Among those who selected “Other” (8.2%), the largest subgroups included Southeast Asian ethnicities, with Hmong (*n* = 9), Laotian (*n* = 8), Cambodian (*n* = 7), Indonesian (*n* = 7), and Thai (*n* = 6) being the most represented. Lastly, 46.58% of participants reported being born in the United States.

### Procedures

The AAR measure was developed in a separate, earlier community-engaged pilot study - funded by Children’s Minnesota Hospitals and Clinics (CM) and the University of Minnesota Clinical and Translational Science Institute (UMN-CTSI) - that combined prior validated measures with applied knowledge from community and academic partners. As an initial step, CM and UMN-CTSI researchers began by identifying candidate items from three psychometrically validated measures: the Revised Racial and Ethnic Microaggressions Scale (R-REMS; [14, 31], the Everyday Discrimination Scale (EDS; Williams et al., [45]), and the Index of Race-Related Stress (IRRS; [41, 42]). These researchers reviewed the item pool and identified items that demonstrated good psychometric properties (e.g., high factor loadings in prior validation studies) and conceptually relevant to AAR during the COVID-19 period, guided both by contemporary reports of hate incidents in the media and lived experiences as Asian Americans. These candidate items were then reviewed in collaboration with community partners from the Coalition of Asian American Leaders (CAAL), a Minnesota-based, community-led social justice and leadership development organization that works across sectors and Asian ethnic communities to advance equity and civic engagement. CAAL partners refined item wording, suggested adaptations to better reflect lived experiences of anti-Asian racism, and proposed additional items based on their direct service and advocacy work in Asian American communities. For example, several subtle racism items (e.g., being told one is “articulate”) and blatant racism items (e.g., being told to “go back to your country”) were adapted from the R-REMS to better reflect the lived experiences of CAAL staff and what their community members are saying. The final item pool consisted of 16 items. Namely, subtle AAR (7 items), items were selected based on psychometric validation studies of the R-REMS, prioritizing items with high factor loadings (≥ 0.5) and deemed relevant by both academic and community partners. For blatant AAR (5 items), items from the EDS were used in conjunction with input from community members about specific AAR experiences, such as being told one does not belong in the United States and being physically threatened for being Asian. Finally, 4 items reflecting cultural AAR were adapted from the IRRS cultural racial discrimination subscale, with academic and community partners selecting items commonly reported by Asian Americans during COVID-19 (e.g., seeing Asians negatively portrayed online or in the media [11, 50]. See Table 1 for the full list of the 16 items and their descriptive statistics (e.g., central tendency, variability).

### Measures

#### Anti-Asian Racism (AAR)

AAR was measured using 16-items that reflects the frequency of subtle (7-items), blatant (5-items), and cultural racism-related experiences (4-items; see Table 1). The 16-items demonstrated good internal consistency (McDonald’s Omega [ω] = 0.95), and response options for all items ranged from 0 (*Never*) to 5 (*Almost Every Day*). For subtle racial discrimination, 7 items adapted from the R-REMS were related to racial microaggressions and demonstrated good internal consistency in our sample (ω = 0.90; [14]). Sample items include, “Someone assumed I wouldn’t be ‘articulate’ or speak well because I am Asian” and “Someone told me that all people in my racial group(s) look alike.” For blatant racial discrimination, 5 items adapted from the EDS [46] were related to verbal and physical harassment and demonstrated good internal consistency (ω = 0.91). Sample items included “I was physically attacked because of my Asian background” and “I was told that I don’t belong in the United States because of my Asian background.” Lastly, for cultural racism, 4 items were adapted from the IRRS [41] and these items pertained to seeing Asians portrayed negatively by politicians or in textbooks, online, or in the mainstream media (ω = 0.91).

To following measures assessed the concurrent validity of the brief AAR measure:

##### Racism Safety Concerns:

A single item was used to evaluate safety concerns as a result of racism (i.e., “In the past 12 months, I have been worried about my safety because of my Asian background). Response options ranged from 0 (*Strongly Disagree*) to 4 (*Strongly Agree*). This single item was developed by our community partners.

##### Depressive Symptoms (PHQ-2):

To assess depressive symptoms, we used a psychometrically validated two-item scale from the Patient Health Questionnaire (PHQ-2; [28]): (1) “Little interest or pleasure in doing things” and (2) “Feeling down, depressed, or hopeless.” Response options ranged from 0 (*Not at all*) to 3 (*Nearly Every day*).

##### Anxiety Symptoms (GAD-2):

To assess anxiety symptoms, we used two-items from the original Generalized Anxiety Disorder-7 scale (GAD-2; [35]): (1) “Feeling nervous, anxious, or on edge” and (2) “Not being able to stop or control worrying.” Response options ranged from 0 (*Not at all*) to 3 (*Nearly Every day*).

##### Firearm Purchasing:

A single item was used to assess whether the participant purchased a firearm in the past year: “In the past 12 months, have you or someone in your home purchased a gun?” A binary response option was used (i.e., 0 = No; 1 =Yes).

## Analytic Approach

Descriptive statistics were calculated to examine item-level mean, standard deviation, and the percentage of exposure for each item. To assess the dimensionality of the AAR measure, we conducted a factor analysis of all the items and began by evaluating eigenvalues for each latent factor, a scree plot, and the percentage of total item variance explained by each latent factor. Of note, an eigenvalue represents the amount of total variance in the observed items accounted for by a given latent factor; thus, factors with larger eigenvalues explain more shared variance among items and are considered more substantively meaningful in determining dimensionality. If the first eigenvalue explained the majority of the total item variance, we investigated a one-factor (i.e., unidimensional) model. Factor loadings for each item were examined to determine the percentage of item-level variance explained by the latent factor, along with model fit. For instance, a standardized factor loading of 0.5 indicates that the latent factor explains 25% of the variance in that item (i.e., 0.50^2^). Model fit was deemed acceptable if the Root Mean Square Error of Approximation (RMSEA) was ≤ 0.06, the Confirmatory Factor Index (CFI) and Tucker-Lewis Index (TLI) were ≥ 0.90, and the Standardized Root Mean Squared Residual (SRMR) was ≤ 0.08 [23]. These fit indices collectively indicate how well the estimated factor model reproduces the observed variance and covariance structure among the items, with values in the recommended ranges suggesting that the model adequately represents the data.

If the one-factor model did not fit the data well, we estimated a confirmatory bi-factor model consistent with the multidimensional conceptualization of the AAR measure, which included three subtype factors: subtle, blatant, and cultural racism. The model fit for the bi-factor model was evaluated using the same criteria as the one-factor model. Additionally, as outlined by Rodriguez and colleagues [36], we assessed dimensionality indices in the bi-factor model, including the Explained Common Variance (ECV), Percent of Uncontaminated Correlations (PUC), and Omega Hierarchical (OmegaH). The ECV reflects the percentage of common variance explained by either the general factor (i.e., AAR) or each of the subtype factors (i.e., subtle, blatant, or cultural racism). The PUC represents the percentage of bivariate correlations attributable primarily to the general factor. OmegaH for the general factor indicates the percentage of variance attributable to individual differences in the general latent factor, while OmegaH for the subtype factors represents the percentage of reliable variance in subscale scores after accounting for the general factor. Evidence for unidimensionality was defined as ECV ≥ 0.8, PUC ≥ 0.7, and OmegaH (general factor) ≥ 0.8 [36]. All factor analytic models were estimated using the *lavaan* package in R [37].

Next, we estimated a Graded Response Model (GRM; Samejima, 2010) to assess how well each item functions in measuring the underlying construct – that is, we evaluated how effectively each item differentiates between individuals at different levels of the latent trait (i.e., AAR) across the ordered response categories. Namely, this model evaluates several key item-level characteristics: *item fit*, which indicates how well each item aligns with the overall model; *discrimination*, which reflects how effectively an item differentiates between individuals with different levels of the trait being measured; *location (also called difficulty)*, which identifies the point on the scale where an item is most informative; and *local dependence*, which checks for residual correlations. These item-level characteristics informed what items to drop from the measure and which ones to keep for the development of the brief AAR measure. Specifically, we prioritized items with high discrimination parameters, location parameters that spanned a broad range of the AAR latent factor scale, items with adequate fit based on item-level fit indices, and items that demonstrated local independence (i.e., residual correlations with other items are less than 0.20). The fit of the GRM was assessed using the same model fit indices as for the factor analytic models. GRMs were estimated using the *MIRT* package in R [6]. Missing data in *lavaan* and *MIRT* were handled using full information maximum likelihood (FIML).

Following the identification of items for the brief AAR measure, we assessed model fit, examined factor loadings, assessed internal consistency, and evaluated the correlation between the latent factors scores for the brief and original measure. To assess concurrent validity, we tested whether the brief and original 16-item measures demonstrated similar associations with the following outcomes: (1) racism-related safety concerns, (2) depressive symptoms, (3) anxiety symptoms, and (4) firearm purchasing. Ordinary Least Squares (OLS) regression was employed for the first three outcomes, while logistic regression was used to account for the binary nature of the fourth outcome (i.e., firearm purchasing). All models included the respondent’s self-reported age, sex, and income as control variables.

## Results

### Preliminary Analysis

For subtle racism, the most commonly reported experience was “Someone told me that all people in my racial group(s) look alike” (M = 0.82, 42.2% of participants), followed by “Someone told me that they ‘don’t see color’ (race) when they see others” (M = 0.76, 34.4% of participants) and “Someone assumed I wouldn’t be ‘articulate’ or speak well because I am Asian” (M = 0.72, 33.7% of participants). For blatant racism, the most commonly reported experience was “I was told an offensive comment or joke about Asian culture, language, food, or history” (M = 0.68, 33.4% of participants), followed by “I was teased or made fun of because of my Asian background” (M = 0.55, 28.2% of participants) and “I was told that I don’t belong in the United States (example: someone telling you, ‘go back to your country!’) because of my Asian background” (M = 0.41, 21% of participants). Lastly, for items related to cultural racism, the most commonly reported experience was “I have seen videos online (example: YouTube, Instagram) that portray Asians in a negative way” (M = 1.08, 49.7% of participants), followed closely by “I have seen the media make Asians look bad (example: dirty, suspicious, or rude)” (M = 1.07, 48.8% of participants). See Table 1 for the frequencies and percentages of participants exposed to each of the AAR experiences.

### Dimensionality Assessment

#### Unidimensional Factor Model

**Fig. 1:**
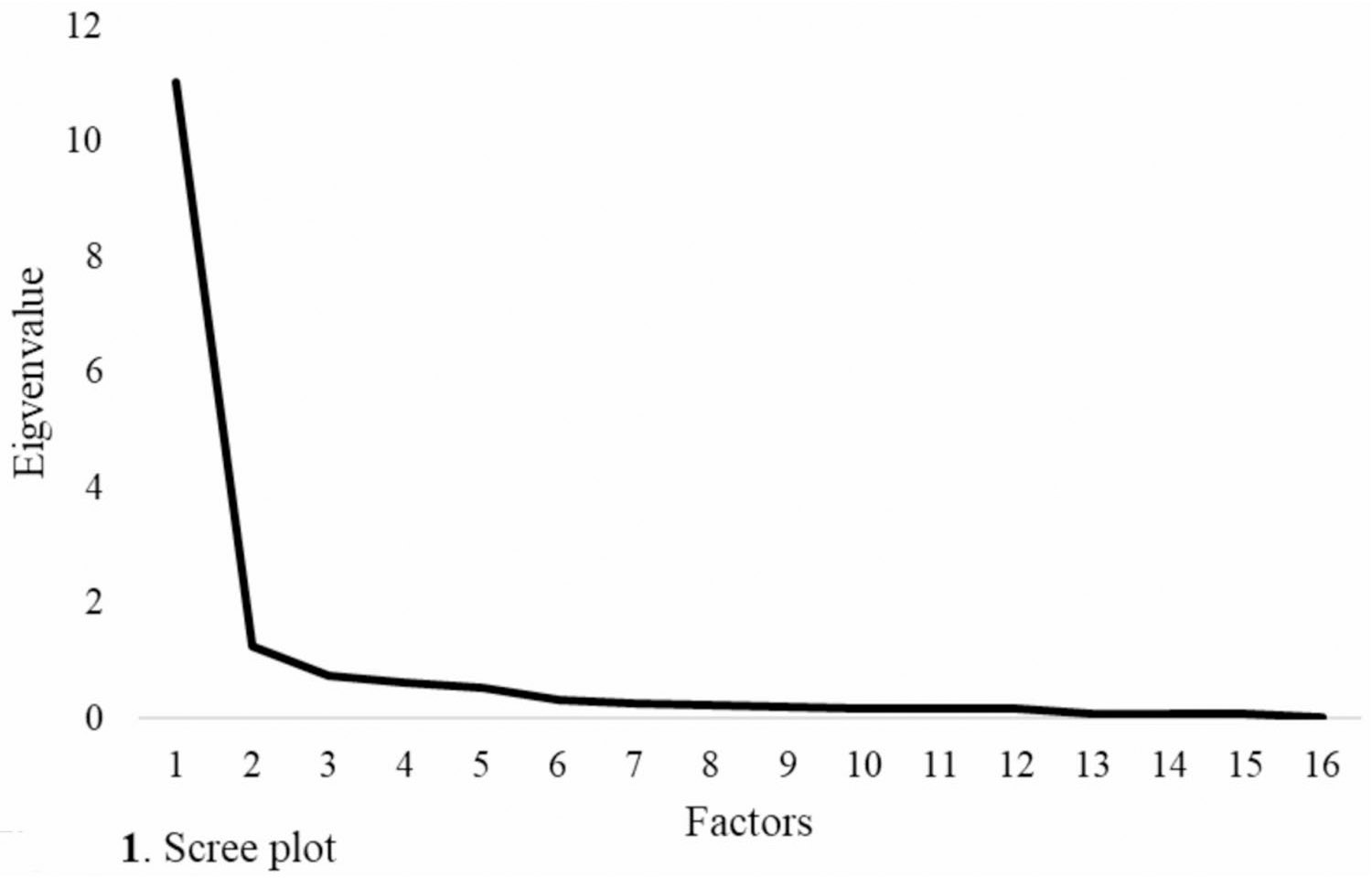
Screen plot

As shown in the scree plot (see Fig. 1), the significant drop in eigenvalue from the first to the second factor suggests that the AAR measure is consistent with a unidimensional factor structure. Additionally, the eigenvalue of the first factor accounted for 69.8% of the total item variance (sum of all eigenvalues) while the second factor would account for an addition 7.8% of variance, further lending evidence that the measure conforms to a unidimensional factor structure. A one-factor model was then estimated; however, this model did not fit the data well (RMSEA = 0.11, CFI = 0.96, TLI = 0.95, SRMR = 0.08). Factor loadings ranged from 0.65 to 0.92 indicating that items, in general, strongly relate to the latent factor – that is, AAR (see Table 2).

#### Bi-Factor Model

We next estimated confirmatory bi-factor models in which all 16 items loaded on to the general factor along with the three subtype factors: 7-items for subtle racism, 5-items for blatant racism, and 4-items for cultural racism. The bi-factor model fit the data well (RMSEA = 0.06, CFI = 0.99, TLI = 0.99, SRMR = 0.03; see Table 2). Upon examining the dimensionality-based indices, however, an ECV of 0.81, PUC of 0.69, OmegaH of 0.88 for the general factor, and OmegaH for the subtype factors (i.e., subtle = 0.10, blatant = 0.002, and cultural = 0.26) indicated that the AAR measure conforms to a unidimensional factor structure. Thus, a unidimensional GRM was estimated to inform the development of the brief AAR measure.

## Graded Response Model

### Item Characteristics and Fit

The unidimensional GRM did not fit the data well, identical to that of the one-factor model. The GRM results, therefore, accentuated the need for further refinement. First, upon investigating item fit, we observed that four items demonstrated poor fit to the data (i.e., RS4, RB2, RC3, and RC4). As a result, these items were not retained for the brief measure. Next, as shown in Table 3, all items exhibited discrimination parameters equal to or greater than 1.54, indicating that all items can effectively differentiate individuals across different levels AAR – i.e., the latent score of AAR. Furthermore, the range of location parameters for each item were relatively high (ranging from 1 to 3), suggesting that most items are measuring severe indices of AAR. The pattern of findings, when taken together, underscores the need for a subset of items that not only differentiates individuals well across the latent continuum (i.e., items with high discrimination) but also captures variation across moderate to high levels of Anti-Asian Racism (i.e., location parameters situated at lower latent scores).

### Local Dependence

We examined local dependence using Chen’s Q_3_ statistics to identify residual correlations [8]. Following the recommendation of Chen and Thissen [8], a Q_3_ statistic of equal to or greater than 0.2 indicated local dependence between item pairs (i.e., item redundancy). Several items within the subtle and blatant racism subscales exhibited local dependence, while all four cultural racism items were locally dependent with one another (see Table 4). Furthermore, no item pairs from the different subtypes of AAR were locally dependent. For locally dependent item pairs, we retained either one item or, in some cases, neither if they exhibited poor fit to the data or were locally dependent on other items.

### Brief Measure Item Selection

The inclusion and exclusion of items for the brief AAR measure were conducted within subtypes. For subtle racism, we excluded RS4 due to poor fit and RS6 and RS7 due to local dependence (see Table 4). While RS2 could have been retained instead of RS1 (a locally dependent item pair), RS1 was selected because RS2 had the lowest discrimination value, indicating lower reliability across the latent score of AAR. Further, we retained RS3 and RS5 because they were more frequently endorsed and had location parameters at moderate levels of AAR, unlike most items, which had location parameters primarily at high levels. Thus, among the subtle racism items, RS1, RS3, and RS5 were retained.

With regards to blatant racism, we excluded RB2 due to poor fit. Of the remaining four blatant racism items, we excluded RB5 rather than RB1 (a locally dependent item pair) as both measures conceptually assessed verbal harassment. While RB5 was more frequently endorsed than RB1, RB1 had location parameters situated at higher levels of the latent score for AAR (see Table 3). Moreover, RB3 and RB4 were locally dependent, and we retained RB4 over RB3 because RB3 was less frequently endorsed and had location parameters only at high levels of AAR. Thus, in the blatant racism subtype, we retained RB1 and RB4.

Lastly, for cultural racism, all four items were locally dependent, and RC3 and RC4 demonstrated poor fit to the data. Moreover, we retained RC1 over RC2 because RC2 specifically addresses cultural racism in online media, while RC1 focuses on media more broadly, making it more universally applicable. That is, Asian Americans who do not regularly use online media platforms (e.g., YouTube, Instagram) may not relate to RC2.

In summary, 6-items were retained for the brief AAR measure: RS1, RS3, RS5, RB1, RB4, and RC1.

## Psychometric Properties of the Brief Measure

**Fig. 2:**
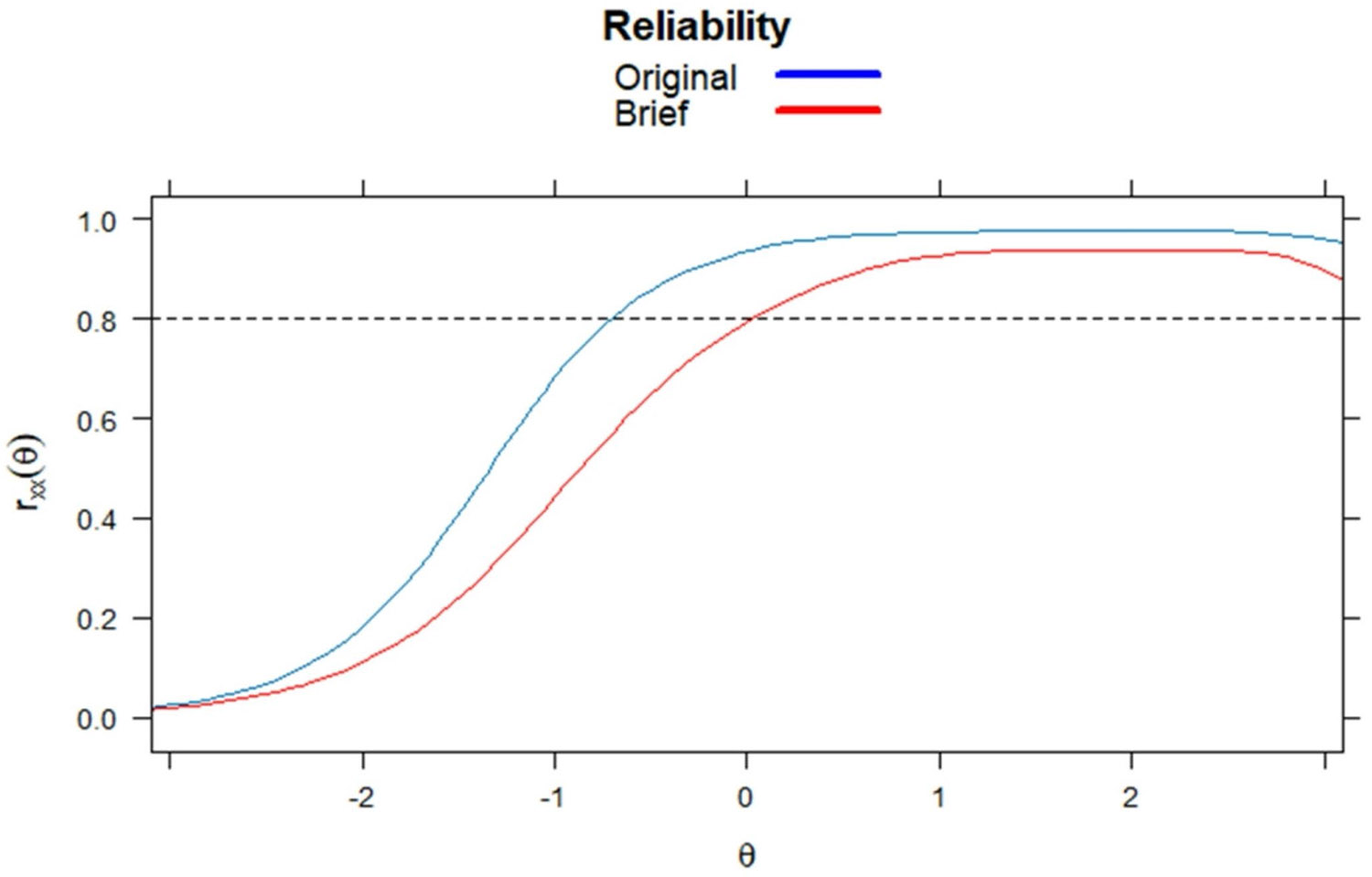
Reliability estimates across the levels of AAR for the original (16-item) and brief (6-item) scale

A unidimensional model for the brief AAR measure fit the data well (RMSEA = 0.06, CFI = 0.99, TLI = 0.99, SRMR = 0.03), with all factor loadings greater than or equal to 0.70, confirming that the latent factor explains 49% or more of the item variances. A unidimensional GRM was estimated, and all items demonstrated comparable discrimination and location parameters as observed in the GRM for the original 16-item scale (see Table 5). Additionally, all items were locally independent. As shown in Fig. 2, the reliability of both measures was comparable at moderate to high levels of AAR, although the original scale exhibited slightly higher reliability at low levels—a by-product of scale truncation (i.e., loss of item information). Finally, a strong positive correlation (*r* = 0.95) was observed between the latent factor scores for the original and brief AAR measure, demonstrating strong convergent validity. The brief measure also demonstrated good internal consistency (ω = 0.87).

## Concurrent Validity

Finally, the original 16-item measure and brief measures of AAR had comparable correlations across the four validity outcomes: (1) racism-related safety concerns, (2) anxiety symptoms, (3) depressive symptoms, and (4) firearm purchasing. As shown in Table 6, the regression coefficients were substantively consistent, with minor variations ranging from 0.001 to 0.049.

## Discussion

AAR is a significant source of stress for Asian Americans. Most measures of AAR fail to capture the diversity of this experience within a single measure or include too many items to be useful in large survey studies measuring multiple constructs. Questionnaires with too many items contribute to respondent burden that often increases the probability of missing data. We developed and psychometrically validated a brief 6-item measure to assess all three types of racism. By adapting items from existing measures of racial discrimination alongside novel items generated by community members during COVID-19, our brief measure efficiently evaluates AAR as a unidimensional construct. Specifically, the items collectively approximate the broader experience of AAR, including subtle, blatant, and cultural racism. This brief measure can be especially useful for real-time data collection (e.g., EMA) or when participants have limited time (e.g., patients in an emergency department). The brief measure can also be beneficial in communitybased participatory research, as it reduces the time burden on both researchers and respondents during data collection. To ensure the brief measure yields valid and reliable scores, items were selected based on high discrimination parameters, a wide range of location parameters, item fit, and local independence. To this end, the brief measure optimizes reliability across different levels of AAR and ensures an accurate assessment of the intended construct.

Consistent with prior research, the brief measure was associated with depressive and anxiety symptoms [7, 21, 43], racism-related safety concerns [51], and firearm purchasing [47]. The magnitude of associations with these outcome variables were comparable between the brief and original measures (see Table 6), demonstrating evidence for concurrent validity. This suggests the brief measure can effectively approximate the psychological toll of AAR. Given the growing reliance on large-scale surveys to investigate health disparities and its mechanisms, brief measures enable researchers to efficiently collect data while making room for the inclusion of additional constructs, including but not limited to important moderators and mediators. Results from the psychometric validation of the brief measure support aggregating the six items in the brief measure to assess AAR as a unidimensional construct, rather than calculating subtype scores (e.g., subtle racism alone). This finding underscores that the 6-items collectively represent an individual’s overall experience of AAR.

It is important to acknowledge that items related to physical attacks were not included in the brief measure. While experiences of physical harassment and attack are severe experiences of blatant racism, and these experiences are less common occurrences relative to other AAR-related experiences within the past 12 months (e.g., 6.6% of participants were physically attacked). Notably, all participants who reported being physically attacked due to being Asian also reported experiencing one or more item in the brief measure. Thus, the purpose of this brief measure is to approximate the frequency of AAR experiences using a concise set of items. Researchers, however, should consider incorporating more severe manifestations of AAR if these are critical to the specific objectives of a study. The primary goal of this measure, however, is to efficiently approximate the everyday experiences of AAR among Asian Americans.

Finally, all items demonstrated satisfactory reliability at moderate to high levels of AAR, but unsatisfactory reliability at low levels of Anti-Asian racism (i.e., when the latent score falls below 0.5). This is likely because racism-related measures often focus on capturing experiences that are able to invoke psychological harms to the victim. Additionally, the latent trait’s skewed distribution—with many reporting no experiences—makes it harder to reliably detect and measure low levels of discrimination, undermining reliability at lower levels of Anti-Asian racism. Future research should consider including items that can reliably differentiate individuals at lower levels of AAR.

### Implications for Clinical Practice

AAR is a pervasive and pernicious stressor for Asian Americans. A brief assessment of AAR offers clinicians and researchers a practical, efficient, and reliable tool to evaluate these experiences. For applied and clinical use, items may be summed to generate a total AAR score. Mental health professionals can use the brief AAR scale to explore subtle, blatant, and cultural forms of racism with clients, thereby enhancing case conceptualization and understanding of how AAR contributes to psychological distress [39, 40]. For instance, if a client reports experiencing blatant interpersonal racism, clinicians may need to engage in safety planning to address immediate risks of harm. In contrast, when clients describe experiences of cultural racism (e.g., omitting the achievements of Asian Americans in US history), clinicians might incorporate exercises that build positive racial identity and encourage participation in affirming environments. Given its brevity, clinical researchers can employ the scale in settings with limited time, such as during EMA or in busy community and clinical settings to better understand the factors that shape Asian American mental health.

### Limitations and Future Directions

Several limitations must be acknowledged and addressed in future studies. First, Asian Americans are not a monolith and AAR experiences may vary across ethnic groups (e.g., Korean or Indian Americans). For example, particularly due to anti-Muslim rhetoric and sentiments, South Asian Americans (e.g., Indian or Pakistani Americans), including both those who are Muslim and those who are merely perceived to be Muslim, may face stigma, discrimination, and hate crimes as part of AAR [38]. While the items in this measure capture racism experiences that may be more general to most Asian Americans (e.g., being told ‘go back to your country!’), researchers need to evaluate whether the items function differently across subgroups—a concept known as differential item functioning. Second, and related to the first limitation, AAR may also be experienced differently by gender, educational attainment, immigration status, and primary language. Although the scale is intended to assess AAR broadly among Asian Americans, we did not formally test measurement invariance or differential item functioning across these subgroups. For instance, Asian American Asian females encounter gendered racism, manifesting as exoticization, hyper-sexualization, and fetishization [13]. Moreover, our sample was predominantly college-educated or higher (unweighted percentage = 87%), and prior work suggests that adults with higher educational attainment tend to report more racial discrimination than those with lower attainment, which limits the generalizability of our findings [15]. Thus, in future work, tests of differential item functioning are needed to ensure that items accurately capture the unique ways racism manifests for both Asian American men and women, as well as across educational attainment levels. Third, an additional limitation is that the survey was administered in English and recruited through an online panel, which may limit representativeness. These design features likely exclude non-English-speaking Asian Americans and individuals without reliable internet access, who may experience AAR differently; future research should prioritize multilingual administration and broader recruitment strategies to enhance coverage. Fourth, the brief measure offers a beginning point evaluating different forms of AAR (e.g., subtle, cultural) to capture the global AAR. However, consistent with the Tripartite Model of Racism [22] and more recent frameworks [19, 32], racism can occur across multiple institutions (e.g., healthcare, education) and be experienced vicariously (i.e., indirect racism). Thus, researchers should continue refining this scale by adding or modifying items to better capture diverse experiences of AAR. Lastly, as is often the case with brief scales, our measure may not fully capture the full scope of AAR, particularly experiences occurring on social media platforms and blatant forms of racism (e.g., being called a racial epithet). For instance, although one item referenced online videos, the retained items primarily reflect broader media portrayals and may not assess interactive, algorithmically amplified, or personally directed harassment within social media platforms. Future research should expand this measure to include items assessing exposure to harassment, hate speech, and emerging forms of AAR (e.g., AI-generated racist content) on social media platforms.

## Conclusion

Despite these limitations, this study developed and validated a brief, unidimensional measure that captures AAR-related experiences across the subtle, blatant, and cultural subtypes. Our findings extend beyond classical test theory approaches and provide a critical foundation for developing a brief, psychometrically sound measure of AAR. Given the need for efficient assessments in research designs, such as EMA, and for populations with limited time to complete long surveys, our measure offers a practical tool for capturing key aspects of AAR without compromising validity or reliability. Future research may refine the scale by assessing other subtypes of AAR. Further psychometric evaluations using contemporary methods, such as moderated nonlinear factor analysis [2, 3], are encouraged to optimize the measure’s reliability and validity across diverse social identities (e.g., gender, ethnicity), ultimately enhancing its generalizability and utility across broader research contexts.

## Figures and Tables

**Table 1:** Item-level descriptive statistics

Subtle Racial Discrimination		M (SD)	% Exposed
RS1	I was told that I should not complain about racism	0.38 (0.93)	17.8%
RS2	Someone told me that they ‘don’t see color’(race) when they see others	0.76 (1.22)	34.4%
RS3	Someone told me that all people in my racial group(s) look alike	0.85 (1.19)	42.2%
RS4	Someone avoided sitting next to me in a public space (e.g., restaurants, movie theaters, subways, buses) because I am Asian.	0.41 (0.92)	20.1%
RS5	Someone assumed I wouldn’t be ‘articulate’ or speak well because I am Asian	0.72 (1.18)	33.7%
RS6	Someone assumed that I would have a lower education because I am Asian	0.39 (0.95)	18.7%
RS7	Someone assumed that I was poor because I am Asian	0.44 (1.05)	19.3%
Blatant Racial Discrimination		M (SD)	% Exposed
RB1	I was teased or made fun of because of my Asian background.	0.55 (1.02)	28.2%
RB2	I was physically threatened because of my Asian background.	0.22 (0.75)	10.6%
RB3	I was physically attacked (example: shoved, punched) because of my Asian background.	0.15 (0.67)	6.6%
RB4	I was told that I don’t belong in the United States (example: someone telling you, ‘go back to your country!’) because of my Asian background.	0.41 (0.94)	21.0%
RB5	I was told an offensive comment or joke about Asian culture, language, food, or history.	0.68 (1.14)	33.4%
Cultural Racial Discrimination		M (SD)	% Exposed
RC1	I have seen the media make Asians look bad (example: dirty, suspicious, or rude)	1.07 (1.30)	48.8%
RC2	I have seen videos online (example: Youtube, Instagram) that portray Asians in a negative way	1.08 (1.32)	49.7%
RC3	I have seen leaders (example: politicians), celebrities, or other prominent figures talk negatively about Asian people	0.96 (1.21)	47.2%
RC4	I have seen Asian ethnic groups (example: Chinese, South Koreans) portrayed negatively in history textbooks, documentaries, or in class	0.80 (1.17)	40.1%

**Table 2:** One-factor and bi-factor model results

Items	One Factor Model	Bifactor Model			
	General Factor	General Factor	Subtle Factor	Blatant Factor	Cultural Factor
RS1	0.789	0.807	0.164		
RS2	0.645	0.657	0.144		
RS3	0.761	0.772	0.16		
RS4	0.81	0.779	0.328		
RS5	0.829	0.775	0.42		
RS6	0.904	0.786	0.537		
RS7	0.907	0.796	0.522		
RB1	0.886	0.896		0.213	
RB2	0.92	0.948		−0.123	
RB3	0.863	0.889		−0.403	
RB4	0.887	0.919		−0.04	
RB5	0.896	0.918		0.282	
RC1	0.853	0.71			0.578
RC2	0.855	0.703			0.582
RC3	0.841	0.722			0.524
RC4	0.822	0.751			0.432

Model fit for the One-Factor Model is RMSEA = 0.11, CFI = 0.96, TLI = 0.95, SRMR = 0.08). Model fit for the Bifactor Model is RMSEA = 0.06, CFI = 0.99, TLI = 0.99, and SRMR = 0.03)

**Table 3:** Graded response model results for the 16-item measure

Items	Discrimination Parameter	Location Parameter 1	Location Parameter 2	Location Parameter 3	Location Parameter 4	Location Parameter 5
RS1	2.542	1.088	1.320	2.096	2.548	3.045
RS2	1.538	0.608	0.975	2.048	2.676	3.136
RS3	2.330	0.250	0.744	1.606	2.236	2.667
RS4	2.567	0.972	1.264	2.080	2.586	3.040
RS5	2.676	0.504	0.834	1.623	2.139	2.444
RS6	3.081	0.965	1.241	1.918	2.383	2.617
RS7	3.135	0.951	1.205	1.692	2.127	2.445
RB1	3.517	0.634	0.992	1.705	2.282	2.649
RB2	4.207	1.258	1.587	1.913	2.375	2.692
RB3	2.679	1.733	2.007	2.348	2.695	2.934
RB4	3.866	0.834	1.248	1.777	2.186	2.514
RB5	3.815	0.478	0.838	1.554	1.993	2.243
RC1	2.452	0.077	0.450	1.356	1.941	2.389
RC2	2.377	0.036	0.465	1.406	1.881	2.424
RC3	2.405	0.114	0.594	1.525	2.173	2.600
RC4	2.569	0.316	0.748	1.681	2.223	2.473

**Table 4:** Local dependence between items

	RM1	RM2	RM3	RM4	RM5	RM6	RM7	RO1	RO2	RO3	RO4	RO5	RC1	RC2	RC3	RC4
RS1	--															
RS2	0.29	--														
RS3			--													
RS4				--												
RS5	0.29				--											
RS6					0.28	--										
RS7					0.27	0.53	--									
RB1								--								
RB2									--							
RB3									0.33	--						
RB4										0.26	--					
RB5								0.28				--				
RC1													--			
RC2													0.51	--		
RC3													0.39	0.37	--	
RC4													0.26	0.27	0.39	--

Blank cells indicate that the residual correlated was < 0.20

**Table 5:** Graded response model and factor analytic model results for brief measure

Items	Discrimination Parameter	Location Parameter 1	Location Parameter 2	Location Parameter 3	Location Parameter 4	Location Parameter 5	Factor Loading
RS1	2.356	1.121	1.370	2.203	2.674	3.172	0.804
RS3	2.436	0.220	0.723	1.606	2.255	2.691	0.802
RS5	2.503	0.492	0.839	1.689	2.245	2.570	0.816
RB1	3.710	0.613	0.984	1.744	2.352	2.720	0.896
RB4	3.615	0.846	1.308	1.884	2.320	2.662	0.895
RC1	1.800	0.056	0.477	1.555	2.271	2.820	0.697

**Table 6:** Convergent Validity Assessment

	Firearm Purchasing		Anxiety Symptoms		Depressive Symptoms		Racial Safety	
			
	b	s.e.	b	s.e.	b	s.e.	b	s.e.
Brief Measure	0.476*	0.081	0.491*	0.067	0.513*	0.072	0.684*	0.059
Original Measure	0.427*	0.073	0.513*	0.068	0.512*	0.065	0.683*	0.054

All four models are adjusted for sex, income, and age
